# Impact of geography and surgical approach on recurrence in global pilonidal sinus disease

**DOI:** 10.1038/s41598-019-51159-z

**Published:** 2019-10-22

**Authors:** Dietrich Doll, Andriu Orlik, Katharina Maier, Peter Kauf, Marco Schmid, Maja Diekmann, Andreas P. Vogt, Verena K. Stauffer, Markus M. Luedi

**Affiliations:** 1 Department of Procto-Surgery, St. Marien-Krankenhaus Vechta, Teaching Hospital of the Hannover University, 49377 Vechta, Germany; 2 Department of Anesthesiology and Pain Medicine, Inselspital, Bern University Hospital, University of Bern, 3010 Bern, Switzerland; 3 Biomedical Statistics PROGNOSIX AG, 8001 Zurich, Switzerland; 4 Sonnenhofspital, Lindenhofgruppe, 3010 Bern, Switzerland

**Keywords:** Gastroenterology, Health services

## Abstract

Pilonidal sinus disease (PSD) is increasing globally. A recent meta-analysis and merged-data analysis showed that recurrence rates in PSD depend essentially on follow-up time and specific surgical procedures. However, the global distribution of surgical approaches and respective recurrence rates have never been studied in PSD. We aimed at studying the impact of geographic distribution of surgical approaches to treat PSD and subsequent geography-specific recurrence rates. We searched relevant databases as described previously. Recurrence rates were then associated with reported follow-up times and geographic origin. We simulated individual patients to enable analogy across data. Globally, recurrence rates range from 0.3% for Limberg/Dufourmentel approaches (95% CI 0.2–0.4) and flaps (95% CI 0.1–0.5) and up to 6.3% for incision (95% CI 3.2–9.3) at 12 months. Recurrence rates range from 0.3% for Karydakis/Bascom approaches (95% CI 0.0–0.8) up to 67.2% for incision (95% CI 7.5–100) in the USA, and 0.0% for primary asymmetric closure in Germany (95% CI 0.0–0.0). Our analysis shows that recurrence rates in PSD not only depend on therapeutic approaches and follow-up time but also on geography. Primary asymmetric closure and various flap techniques remain superior regardless of the geographical region. Some approaches have extraordinarily good outcomes in specific countries.

## Introduction

The incidence of pilonidal sinus disease is increasing globally. About 100/100,000 inhabitants per year are affected in Germany^1^, with even higher numbers reported for Turkey^2^. Since 2013, American, German and Italian societies have published guidelines on best clinical practice^3–5^. While debate over the disease’s etiology and pathomechanisms is ongoing^6–9^, we showed recently that recurrence rates in PSD depend essentially on follow-up time and the specific surgical procedure used^10^. In a meta-analysis and merged-data analysis of surgical treatment options, follow-up times, and recurrence rates in 89,583 patients, recurrence after Limberg/Dufourmentel operations was as low as 0.6% at 12 months and 1.8% at 24 months postoperatively. Recurrence after Karydakis/Bascom procedures was 0.2% (95% CI 0.1–0.3%) at 12 months and 0.6% (95% CI 0.5–0.8%) at 24 months postoperatively. Primary midline closure after 240 months was associated with recurrence rates of 67.9% (95% CI 53.3–82.4%)^10^ (Table 1).

**Table 1:** Recurrence rates (RR) in different surgical approaches deriving from including all available studies from all geographical regions for 12, 24, 60, and 120 months follow-up time.

Surgical method (total patients included)	12 months		24 months		60 months		120 months		Citations
	RR in % (95% CI)	Nr. at risk	RR in % (95% CI)	Nr. at risk	RR in % (95% CI)	Nr. at risk	RR in % (95% CI)	Nr. at risk	
Primary open (6351)	1.3 (1.0–1.7)	5715	4.2 (3.6–4.8)	3496	13.9 (12.2–15.6)	1222	28.1* (23.9–32.4)	241*	^20– 122^
Primary median closure (15011)	3.1 (2.8–3.5)	12,484	8 (7.4–8.6)	6956	14.9 (13.8–15.9)	2505	27.9 (25.5–30.4)	631	^20, 24, 27, 29, 31, 34– 38, 44, 46, 48, 52, 54, 56, 57, 59, 61– 66, 69, 73– 78, 81– 84, 86, 89– 91, 93– 100, 106– 108, 115, 118, 122– 248^
Primary asymmetric closure (2538)	0.6 (0.3–0.9)	2432	1.1 (0.7–1.6)	1807	2.7 (1.9–3.6)	1205	6.3* (4.8–7.7)	937*	^65, 67, 100, 129, 183, 192, 249– 263^
Karydakis/Bascom (6276)	0.5 (0.3–0.7)	5868	1.6 (1.2–2.0)	3630	6.3 (5.0–7.5)	853	NA	NA	^16, 21, 29, 41, 49, 66, 78, 88, 133, 163, 172, 184, 189, 192, 195, 199, 200, 202, 262– 316^
Limberg/Dufourmentel (11470)	0.3 (0.2–0.4)	10,937	1.5 (1.2–1.7)	7527	5.9* (5.1–6.7)	1638*	NA	NA	^25, 28, 31, 33, 52, 56, 67, 92, 100, 127, 128, 130, 131, 137, 139, 140, 148, 149, 163, 165, 170, 172, 174, 182, 190– 192, 201, 205, 207, 208, 231– 233, 243, 246, 267, 268, 270, 272– 274, 291, 292, 295, 297, 305– 309, 311– 313, 317– 398^
Marsupialization (1896)	2.2 (1.4–2.9)	1253	5.7 (4.2–7.1)	800	7.8* (5.9–9.6)	566*	NA	NA	^30, 37, 46, 60, 65, 69, 71, 77, 78, 89, 97, 104, 110, 137, 152, 156, 175, 207, 208, 332, 399– 416^
Limited excision (3346)	5.1 (4.2–6.0)	2746	7.4 (6.2–8.5)	1810	13.1 (11.1–15.0)	804	NA	NA	^31, 34, 52, 60, 61, 69, 75, 82, 87, 96, 98, 103, 112, 116, 136, 138, 170, 187, 212, 220, 229, 241, 290, 299, 311, 344, 399, 414, 417– 436^
Pit picking (5432)	2.8 (2.4–3.3)	5221	6.6* (5.9–7.4)	1962*	14.2 (12.5–15.9)	1401	NA	NA	^16, 22, 55, 69, 109, 193, 216, 278, 298, 309, 437– 452^
Flaps (3073)	0.3 (0.1–0.5)	2902	1.1* (0.7–1.5)	1687*	6.4* (4.9–8.0)	708*	NA	NA	^91, 98, 100, 108, 135, 173, 185, 191, 193, 227, 309, 322, 343, 362, 369, 375, 379, 424, 434, 453– 488^
Incision and drainage (293)	6.3* (3.2–9.3)	259*	22.3 (15.8–28.9)	243	36.8* (26.3–47.2)	121*	NA	NA	^20, 52, 55, 77, 184, 212, 223, 253, 489^
Phenol only (1453)	1.6* (0.9–2.3)	1244*	14.7 (12.1–17.3)	687	NA	NA	NA	NA	^53, 76, 114, 242, 363, 385, 490– 505^
Laser treatment (77)	2.2* (0.0–4.8)	74*	11.8* (0.8–22.9)	47*	NA	NA	NA	NA	^506– 508^
Others (1363)	2.8 (1.9–3.8)	1247	6.6* (4.8–8.4)	624*	19.8 (15.7–23.8)	498	NA	NA	^15, 16, 309, 430, 489, 509– 523^
Partial closure (202)	0.6* (0.0–1.4)	202*	1.1* (0.0–2.5)	201*	11.2* (5.9–16.6)	161*	NA	NA	^62, 98, 143^

Geography – and thereby specific genetic mechanisms, healthcare settings and socioeconomic factors – has been shown to affect manifold diseases, and must be considered when studying a disease worldwide^11,12^. However, the global distribution of surgical approaches and the geography of recurrence rates have never been assessed in PSD, one of the most frequent surgical diagnoses^1^.

We therefore studied the impact of geographic distribution of surgical approaches on the treatment of PSD and the associated recurrence rates in order to evaluate the quality of selected procedures. We used our previously established database of publications from the years 1833–2017 to study PSD treatment strategies, follow-up time, and country of origin^10^. We hypothesized that geography affects recurrence rates in PSD and an extensive analysis of data will allow specific recommendations for different geographic regions.

## Results

Our original search and processing strategy was described previously^10^. In brief, after exclusion of duplicates, we screened 5,768 studies across various databases. Data pertaining to malignancies, embryonic development, and body regions other than the presacral intergluteal location was additionally excluded, leaving 1,148 articles. Of these, 408 reports lacked data on follow-up time or on recurrence or both. Subsequently, data deriving from 740 studies was included in the merged data analysis^10^. The flow chart based on the preferred reporting items for systematic reviews and meta-analyses (PRISMA) can be found in our first study; the same applies for the heterogeneity analysis of the data involved^10^.

**Figure 1:**
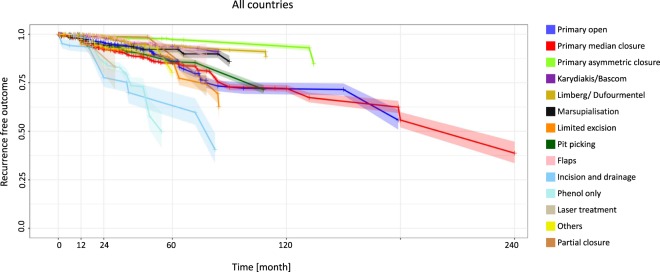
All countries: Kaplan-Meier-estimator depicting recurrence free outcome of the study population as a function of follow-up time. The data used include all available studies from all geographical regions. 95% confidence intervals are shown by shaded lines. The number of patients at risk for 12, 24, 60, and 120 months follow-up time are shown in Table 1.

Looking at the overall results, the recurrence rates at 12 months ranged from 0.3% for the Limberg/Dufourmentel approach (95% CI 0.2–0.4) and the flaps approach (95% CI 0.1–0.5) to 6.3% for the incision and drainage approach (95% CI 3.2–9.3). At 60 months, the incision and drainage approach showed the highest recurrence rate: 36.8% (95% CI 26.3–47.2) (Fig. 1, Table 1).

**Figure 2:**
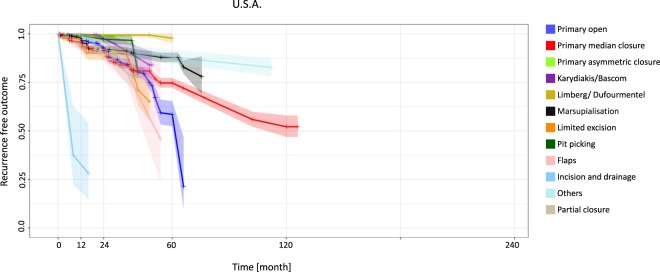
United States: Kaplan-Meier estimator depicting recurrence-free outcome of the study population as a function of follow-up time. The data used include all available studies from the United States. 95% confidence intervals are shown by shaded lines. The number of patients at risk of recurrence at 12, 24, 60, and 120 months of follow-up is shown in Table 2.

In the United States at 12 months the recurrence rates ranged from 0.3% for the Karydakis/Bascom approach (95% CI 0.0–0.8) to 67.2% for the incision and drainage approach (95% CI 7.5–100). The Limberg/Dufourmentel approach showed the lowest recurrence rate at 60 months, with 2.3% (95% CI 0.0–4.9) (Fig. 2, Table 2).

**Table 2:** Recurrence rates (RR) in different surgical approaches deriving from including available studies from the U.S.A. for 12, 24, 60, and 120 months follow-up time.

USA	12 months		24 months		60 months		120 months		Citations
Surgical method (total patients included)	RR in % (95% CI)	Nr. at risk	RR in % (95% CI)	Nr. at risk	RR in % (95% CI)	Nr. at risk	RR in % (95% CI)	Nr. at risk	
Primary open (2124)	2.2 (1.5–2.8)	1708	7.4 (5.7–9.1)	734	41.5 (30.2–52.8)	69	NA	NA	^21, 37, 41, 43, 46, 57, 62, 63, 69, 77, 79, 104, 105, 107, 108, 111, 524– 540^
Primary median closure (3650)	4.3 (3.6–5.1)	2754	8.5 (7.4–9.7)	2119	25.3 (21.9–28.7)	388	47.9 (37.1–58.6)	44	^37, 46, 57, 62, 63, 69, 77, 107, 108, 152, 156, 157, 170, 175, 181, 185, 227, 237, 525– 529, 532, 533, 538, 540– 559^
Primary asymmetric closure (176)	0.6* (0.0–1.7)	170*	0.6 (0.0–1.7)	160	NA	NA	NA	NA	^257, 560, 561^
Karydakis/Bascom (236)	0.3* (0.0–0.8)	236*	2.4* (0.2–4.7)	160*	NA	NA	NA	NA	^21, 41, 562– 565^
Limberg/Dufourmentel (164)	0.6* (0.0–1.8)	156*	0.6* (0.0–1.8)	145*	2.3 (0.0–4.9)	120	NA	NA	^170, 351, 374, 566^
Marsupialization (1475)	2.4 (1.4–3.3)	933	8.0* (6.0–10.1)	526*	12.1* (9.2–15.0)	332*	NA	NA	^37, 46, 69, 77, 104, 152, 156, 175, 399– 401, 404, 405, 410, 411, 416, 527, 532, 538, 549, 567– 571^
Limited excision (780)	5.8 (3.3–8.3)	301	10.1* (6.0–14.2)	162*	NA	NA	NA	NA	^69, 170, 399, 430, 435, 527, 535, 572– 578^
Pit picking (328)	0.9 (0.0–2.0)	328	2.8 (0.6–4.9)	213	NA	NA	NA	NA	^69, 445, 579, 580^
Flaps (595)	6.9 (4.6–9.3)	517	10.7* (7.0–14.4)	176*	NA	NA	NA	NA	^108, 185, 227, 485, 527, 533, 581– 583^
Incision and drainage (24)	67.2* (7.5–100)	16*	NA	NA	NA	NA	NA	NA	^77, 528^
Others (374)	0.9* (0.0–1.9)	364*	8.2* (4.7–11.7)	237*	11.9* (7.5–16.3)	234*	NA	NA	^430, 510, 520, 584, 585^
Partial closure (215)	5.5* (2.2–8.8)	199*	8.9* (4.5–13.3)	168*	NA	NA	NA	NA	^62, 526, 528, 533, 549, 586^

**Figure 3:**
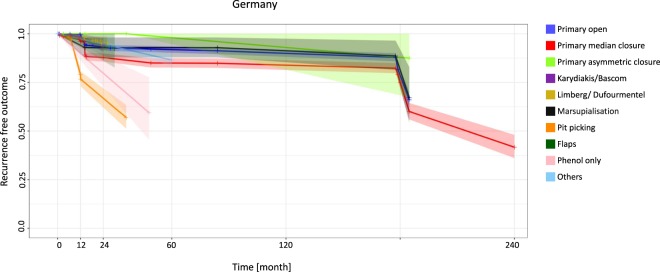
Germany: Kaplan-Meier estimator depicting recurrence-free outcome of the study population as a function of follow-up time. The data used include all available studies from Germany. 95% confidence intervals are shown by shaded lines. The number of patients at risk of recurrence at 12, 24, 60, and 120 months of follow-up is shown in Table 3.

In Germany, patients undergoing the primary asymmetric closure approach had no recurrence at 12 months (95% CI 0.0–0.0), whereas patients undergoing the pit-picking approach had a much higher recurrence rate of 21.0% (95% CI 16.7–25.3). At 120 months four surgical approaches showed recurrence below 20%: the primary open approach (10.0%) (95% CI 7.9–12.1), the primary median closure approach (16.1%) (95% CI 13.1–19.2), the primary asymmetric closure approach (7.1%) (95% CI 0.0–22.1) and the marsupialization approach (8.8%) (95% CI 2.0–15.5) (Fig. 3, Table 3).

**Table 3:** Recurrence rates (RR) in different surgical approaches deriving from including all available studies from Germany for 12, 24, 60, and 120 months follow-up time.

Surgical method (total patients included)	12 months		24 months		60 months		120 months		Citations
	RR in % (95% CI)	Nr. at risk	RR in % (95% CI)	Nr. at risk	RR in % (95% CI)	Nr. at risk	RR in % (95% CI)	Nr. at risk	
Primary open (1457)	0.5 (0.1–0.8)	1263	6.9* (5.3–8.5)	851*	8.1* (6.3–9.9)	752*	10* (7.9–12.1)	706*	^13, 26, 44, 54, 59, 64– 66, 92, 97, 115, 117, 122, 587, 588^
Primary median closure (1320)	3.9 (2.7–5.1)	1043	12.2 (9.8–14.6)	655	15.0* (12.2–17.9)	554*	16.1* (13.1–19.2)	507*	^13, 44, 54, 59, 64– 66, 97, 115, 122, 123, 180, 211, 588– 590^
Primary asymmetric closure (87)	0.0* (0.0–0.0)	48*	0.0* (0.0–0.0)	33*	2.0* (0.0–6.3)	26*	7.1* (0.0–22.1)	17*	^65, 588, 591, 592^
Karydakis/Bascom (332)	1.5* (0.0–3.2)	226*	5.9 (2.0–9.8)	151	NA	NA	NA	NA	^66, 298, 587^
Limberg/Dufourmentel (434)	1.9* (0.5–3.4)	278*	5.1* (1.2–8.9)	54*	NA	NA	NA	NA	^92, 320, 357, 366, 394, 593– 595^
Marsupialization (98)	6.0* (1.3–10.6)	98*	7.1* (1.5–12.7)	93*	7.1* (1.5–12.7)	76*	8.8* (2.0–15.5)	57*	^13, 65, 97, 588^
Pit picking (676)	21 (16.7–25.3)	553	33.1* (25.2–41.1)	158*	NA	NA	NA	NA	^298, 441, 447, 452^
Flaps (26)	3.1* (0.0–7.6)	26*	6.2* (0.0–15.2)	26*	NA	NA	NA	NA	^596^
Phenol only (37)	10.1* (3.3–16.9)	37*	20.3* (6.7–33.8)	37*	NA	NA	NA	NA	^497^
Others (498)	2.7* (2.0–3.4)	498*	5.4* (4.0–6.8)	498*	13.5 (9.9–17.0)	498	NA	NA	^513^

**Figure 4:**
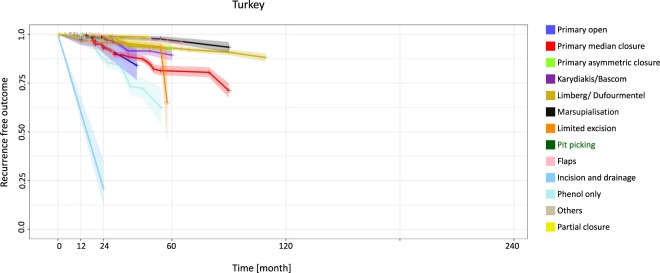
Turkey: Kaplan-Meier estimator depicting recurrence-free outcome of the study population as a function of follow-up time. The data used include all available studies from Turkey. 95% confidence intervals are shown by shaded lines. The number of patients at risk of recurrence at 12, 24, 60, and 120 months of follow-up is shown in Table 4.

Patients in Turkey had very good outcomes with the flaps approach at 12 months (0.0% recurrence) (95% CI 0.0–0.0), but the incision and drainage approach was associated with recurrence of 39.4% at 12 months (95% CI 12.7–66.2) and 78.8% (95% CI 25.3–100) at 24 months (Fig. 4, Table 4).

**Table 4:** Recurrence rates (RR) in different surgical approaches deriving from including all available studies from Turkey for 12, 24, 60, and 120 months follow-up time.

Surgical method (total patients included)	12 months		24 months		60 months		120 months		Citations
	RR in % (95% CI)	Nr. at risk	RR in % (95% CI)	Nr. at risk	RR in % (95% CI)	Nr. at risk	RR in % (95% CI)	Nr. at risk	
Primary open (143)	2.7* (0.0–5.5)	143*	5.9* (1.4–10.4)	120*	NA	NA	NA	NA	^32, 114, 118^
Primary median closure (2902)	0.8 (0.4–1.1)	2818	7.0* (5.7–8.2)	1383*	18.8* (15.7–21.8)	417*	NA	NA	^118, 127, 133, 136, 137, 139, 143, 148, 149, 158– 160, 169, 172– 174, 183, 192, 199– 201, 232, 242, 243, 246, 247, 597– 603^
Primary asymmetric closure (727)	1.7 (0.7–2.8)	621	2.5 (1.1–3.8)	403	7.4 (4.4–10.4)	257	NA	NA	^183, 192, 253, 259, 261, 262^
Karydakis/Bascom (2471)	0.8 (0.4–1.1)	2356	1.7* (1.1–2.4)	1343*	10.7 (7.4–13.9)	257	NA	NA	^133, 172, 192, 199, 200, 262, 264, 267, 268, 270, 273, 274, 276, 279, 282, 284, 289– 292, 295, 296, 300, 305– 309, 603^
Limberg/Dufourmentel (7653)	0.1 (0.0–0.2)	7369	1.1 (0.8–1.4)	5624	7.1* (5.9–8.2)	988*	NA	NA	^127, 137, 139, 148, 149, 172, 174, 192, 201, 232, 243, 246, 267, 268, 270, 273, 274, 291, 292, 295, 305– 309, 317, 319, 321– 323, 327, 329, 330, 332, 338– 340, 342– 344, 346– 350, 352– 355, 359, 362– 364, 368, 369, 371– 373, 375, 379, 382, 385, 387, 395, 398, 600, 603– 607^
Marsupialisation (728)	0.4 (0.0–0.9)	688	1.3* (0.4–2.3)	419*	3.1* (1.4–4.7)	358*	NA	NA	^137, 332, 402, 403, 415, 598, 599, 603, 606^
Limited excision (1402)	3.1 (2.2–4.1)	1402	4.1* (2.8–5.5)	423*	NA	NA	NA	NA	^136, 290, 344, 417, 420, 424, 428, 429, 608^
Pit picking (204)	0.4* (0.0–1.0)	204*	0.8* (0.0–2.0)	204*	NA	NA	NA	NA	^309^
Flaps (2262)	0 (0.0–0.0)	2219	0.4* (0.1–0.8)	1292*	3.8* (2.5–5.2)	670*	NA	NA	^173, 309, 322, 343, 362, 369, 375, 379, 424, 454– 456, 458, 459, 461, 462, 464, 466, 469– 471, 473, 475, 476, 478– 481, 599^
Incision and drainage (52)	39.4* (12.7–66.2)	52*	78.8 (25.3–100)	52	NA	NA	NA	NA	^253^
Phenol only (1089)	0.5 (0.1–0.9)	990	12.6 (9.9–15.3)	607	NA	NA	NA	NA	^114, 242, 363, 385, 490– 496, 498, 504, 505, 609^
Others (103)	2.6* (0.0–5.2)	103*	5.3* (0.1–10.4)	56*	NA	NA	NA	NA	^309, 509, 521^
Partial closure (73)	0.3* (0.0–1.1)	73*	0.7* (0.0–2.1)	73*	NA	NA	NA	NA	^143^

**Figure 5:**
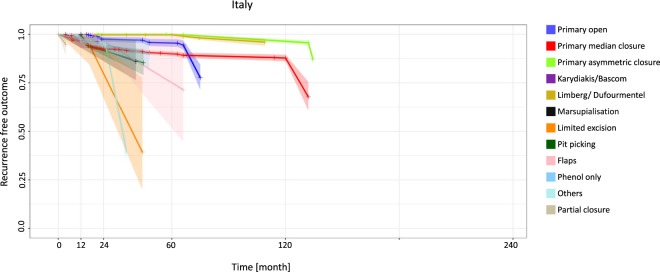
Italy: Kaplan-Meier estimator depicting recurrence-free outcome of the study population as a function of follow-up time. The data used include all available studies from Italy. 95% confidence intervals are shown by shaded lines. The number of patients at risk of recurrence at 12, 24, 60, and 120 months of follow-up is shown in Table 5.

Italy delivered outstanding results at 12 months for four procedures: recurrence was 0.0% for the primary open approach (95% CI 0.0–0.0), the primary asymmetric closure approach (95% CI 0.0–0.0), the Limberg/Dufourmentel approach (95% CI 0.0–0.0) and the flaps approach (95% CI 0.0–0.0). Recurrence was relatively low with the marsupialization approach at 12 months (4.1%) (95% CI 0.5–7.7) and with the primary asymmetric closure approach at 120 months (3.6%) (95% CI 2.4–4.8) (Fig. 5, Table 5). Similar specifics can be shown for additional countries and regions such as Australia, New Zealand, Greece and Asia (Supplemental Figs 1–3, Supplemental Tables 1–3).

**Table 5:** Recurrence rates (RR) in different surgical approaches deriving from including all available studies from Italy for 12, 24, 60, and 120 months follow-up time.

Surgical method (total patients included)	12 months		24 months		60 months		120 months		Citations
	RR in % (95% CI)	Nr. at risk	RR in % (95% CI)	Nr. at risk	RR in % (95% CI)	Nr. at risk	RR in % (95% CI)	Nr. at risk	
Primary open (1243)	0 (0.0–0.0)	1203	2.5* (1.2–3.8)	441*	4.5* (2.5–6.6)	267*	NA	NA	^35, 56, 70, 72, 80, 91, 94– 96, 99, 100, 120, 610– 613^
Primary median closure (5583)	3.3 (2.8–3.8)	4184	7.6 (6.7–8.5)	1939	10.1* (8.8–11.4)	786*	12.2 (10.3–14.2)	382	^35, 56, 91, 94– 96, 99, 100, 129, 144, 146, 161, 162, 165– 168, 171, 179, 182, 186, 197, 205, 209, 214, 215, 218– 222, 610– 621^
Primary asymmetric closure (1099)	0.0* (0.0–0.0)	1099*	0.0* (0.0–0.0)	1096*	0.1* (0.0–0.2)	1022*	3.6* (2.4–4.8)	937*	^100, 129, 256, 260, 622^
Karydakis/Bascom (109)	3.7 (0.0–7.4)	109	NA	NA	NA	NA	NA	NA	^16, 622^
Limberg/Dufourmentel (944)	0.0* (0.0–0.0)	944*	0.3* (0.0–0.7)	891*	0.3 (0.0–0.7)	618	NA	NA	^56, 100, 165, 182, 205, 328, 334, 336, 623^
Marsupialization (43)	4.1* (0.5–7.7)	43*	8.2* (1.0–15.4)	43*	NA	NA	NA	NA	^406^
Limited excision (18)	4.0* (0.0–12.4)	18*	20.4* (0.0–47.3)	16*	NA	NA	NA	NA	^96, 220^
Pit picking (2508)	0.3 (0.1–0.5)	2508	8.4* (5.4–11.3)	1765*	NA	NA	NA	NA	^16, 439, 444, 451^
Flaps (13)	0.0* (0.0–0.0)	13*	3.6* (0.0–9.6)	12*	25.0* (0.0–66.8)	8*	NA	NA	^91, 100^
Phenol only (68)	2.6* (0.5–4.6)	68*	5.1* (1.0–9.3)	68*	NA	NA	NA	NA	^613^
Others (815)	3.5 (2.1–4.9)	728	5.2* (3.2–7.3)	344*	NA	NA	NA	NA	^16, 182, 511, 514, 516, 523, 624^
Partial closure (58)	NA	NA	NA	NA	NA	NA	NA	NA^612^	^625– 636^

**Figure 6:**
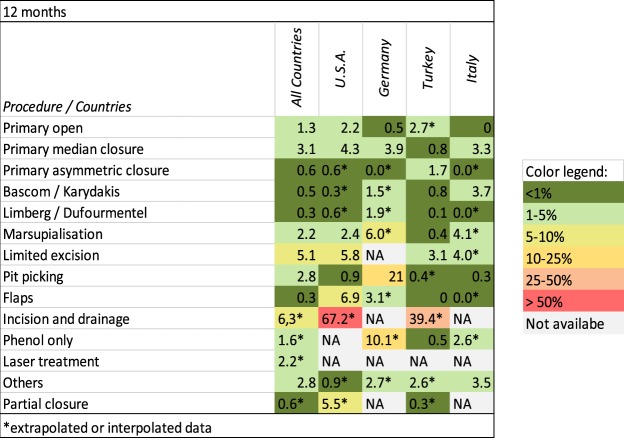
Procedure-specific recurrence rates in PSD [%] are shown at the time point 12 months. Extrapolated or interpolated data are marked with an asterisk (*).

**Figure 7:**
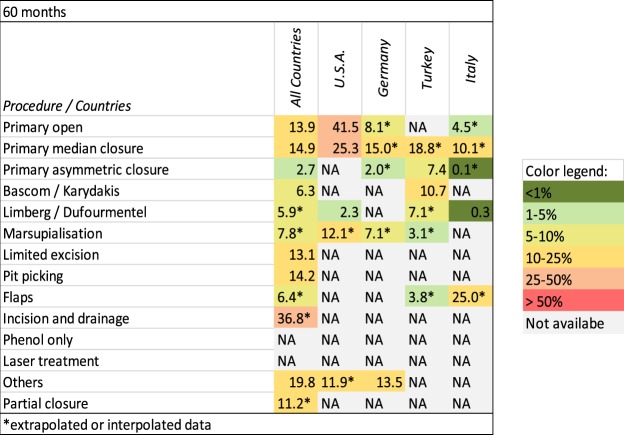
Procedure-specific recurrence rates in PSD [%] are shown at the time point 60 months. Extrapolated or interpolated data are marked with an asterisk (*).

Figures 6 and 7 provide an overview of recurrence rates of all procedures studied after 12 and 60 months in various geographic settings.

## Discussion

We analyzed global data of more than 80,000 PSD patients for the years 1833 to 2017. Whereas in our previous study we only looked at follow-up time dependent recurrence rates of different surgical procedures, we now analyzed the geography’s impact on recurrence rates. Most of the patients analyzed were from the US (8,017), Germany (4,965), Turkey (19,809) or Italy (12,443). Focusing on the surgical approaches used in different countries and regions of the world, we assessed the recurrence rates at different follow-up times and found a correlation between geography and PSD recurrence for a variety of surgical treatments.

Not all surgical approaches were used in all the countries analyzed, and certain preferences exist in the choice of surgical approach in each country, leading to differences in recurrence rates of specific surgical approaches between the geographical regions. Because not all nations and continents report PSD patient treatments in sufficient numbers, some geographical regions had to be excluded to ensure sufficient data quality. For example, countries in Africa, Asia and South America were not included due to missing data, and/or a very low disease burden.

The merged data analysis is potentially less powerful than a systematic review consisting entirely of randomized controlled trials (RCT’s). Many of the studies we cited could have qualified as RCT’s, but our approach enabled inclusion of enough patients to be able to compare all the surgical methods available in different geographical regions. Certain surgical approaches are not being used for treatment in all the analyzed geographical regions. This lack of data provides important information about established treatments in specific health care settings and geographic regions respectively. The patient number or number at risk also differs strongly between regions, probably pointing out specific economic and clinical peculiarities. Also, abrupt drops and interpolations in our figures need to be interpreted with caution because the curves about recurrence rates are influenced by the cohort sizes of underlying studies: The methodologies of the underlying studies may indirectly create a certain bias.

The primary open approach showed insufficient success in most geographical regions, with a recurrence rate of 41.5% being observed at 60 months in the US. This has not been shown before, and is appalling in its magnitude. Reasons for the high recurrence rate are not yet evident. Current evidence implies that non healing beyond 6 month post-surgery should be considered as recurrent disease^13,14^. The application of metronidazole 10% ointment has enabled a faster wound closure in patients^15^ and healing can be regularly expected within 6 months, however, some surgical wounds only close after 9 months or later. Further, non-healing wounds must not be confused with a scar overlying the sacral bone. By definition recurrent PSD is defined as a new sinus tract. Although stringent criteria to distinguish healing disorders versus recurrence are available, some variation in recurrence rate observation between countries might unfortunately have been published.

The primary midline closure, which is not recommended for use anymore, showed a recurrence rate of 25.3% at 60 months in the USA. The pit-picking approach had a high recurrence rate of 33.1% at 24 months in Germany and should therefore be used only selectively for the treatment of minor disease in PSD patients. Nevertheless, interim results of current studies on pit picking are more promising. In contrast to the finding in Germany, pit picking showed a very low recurrence rate of 0.8%* at 24 months in Turkey, justifying this treatment in the Turkish medical setting. Strikingly, the Karydakis/Bascom approach in Greek cohorts showed the lowest observed recurrence rate at the 120-month follow-up. Nevertheless, it should be kept in mind that Karydakis never fully disclosed his data details of several thousands of Greek recruits. In Italy, endoscopic therapy approaches of different names are emerging and first results appear to be very promissing^16^. Given our data base embracing evidence ranging from 1833 to 2017, it is currently too early, however, to comment on their long-term recurrence rate since our data base misses the most recent publications. This approach might become a very promising approach in PSD surgery.

Therapy of open wounds following surgery has not been standardized, and may be performed by either a doctor, a nurse or a family member. This is not sufficiently well described in most of the studies we cited. Even the most recently published US guidelines do not recommend a particular type of wound care for primary open treatment^17^. As increased duration of open wound treatment may increase recurrence rate^14^, and elevated body weight with consecutive metabolic derangements may prolong wound healing, body mass index (BMI) in relation to treatment applied may further influence recurrence rate in primary and secondary treatments.

Obviously, there is some mastery of certain surgical methods which are widely applied in some countries, and this contributes to better regional results. In other countries, the same methods used less often may show more dismal outcomes, contributing to the geographic differences in recurrence rates. Recently, Doll *et al*. have shown that patients with strong axial hair shafts are more prone to pilonidal sinus disease, and Bosche *et al*. found short cut hair less 2 cm length in the pilonidal nests^18,19^ indicating that both genetic disposition and cultural hair styles can contribute to regional variation of pilonidal sinus incidence and recurrence rate.

Furthermore, our current study analyzes results published in scientific journals. These studies are often run at large university hospitals. While industrialized countries have better resources and can document, study and report therapy outcomes, more rural countries with less funding may struggle to do so. In terms of economics, the costs of treatment may differ based on location, treating institution and type of therapy.

Our results allow a more differentiated view of PSD treatment. Surgical approaches should be selected carefully based on treatment efficacy in general, and geographical influences have to be taken into account when aiming for optimal treatment efficacy.

In summary, recurrence rates of different surgical approaches used in the treatment of PSD are influenced by geographical factors. Certain surgical approaches – such as primary asymmetric closure and different flap techniques – remain superior, regardless of the geographical region. This is powerful evidence since the clinical settings, the genetic background of the patient population and economic settings do vary between different countries. Methods such as limited excision and phenol treatment should be limited to selected settings due to their high recurrence rates. Under certain circumstances their use can be justified by the lack of need for a hospital stay or as low-cost variant of treatment. Geographic peculiarities were identified, such as high recurrence rates for the primary open approach in the US, suggesting that other methods should be preferred in the American setting. Pit picking should be selectively applied in Germany due to its high recurrence rate, and ways to improve this interesting minimal invasive procedure should be investigated. The same surgical method is already showing promising results in Turkey. In the future, detailed investigation into geographical differences in recurrence rates for the same surgical PSD method may lead to the identification of further co-factors for recurrence in pilonidal sinus disease. Therefore, the standardized definition of recurrence should uniformly be used^13,14^.

## Methods

Our original search was described previously^10^. In brief, we searched for the NCBI Medical Subject Heading (MeSH) term “pilonid*”, as well as [“cyst” AND “dermoid”] in MEDLINE, Ovid, PubMed Central, PubMed, Scopus, Embase, the Cochrane Central Register of Controlled Trials (CENTRAL) and other search engines to build the PSD database^10^. Publications from 1833 to 2017 in English, French, German, Italian, and Spanish were captured^10^. Reports in other languages were retrieved if recurrence at specific follow-up times and definitive treatment strategies were provided (National Health Service international prospective register of systematic reviews PROSPERO number 42016051588)^10^. Data were organized with Microsoft Excel (Version 2016, Microsoft Corp., Redmond, WA)^10^. Specific surgical approaches described in a report were listed in a data row, while columns included citation details (incl. country of origin), follow-up times, number of patients studied, recurrence, and study details^10^. Regularly recorded information included which hospital(s) participated and which region the patients came from. In the very few studies where this was not obvious, the patient’s country of origin was defined as where the first author’s hospital was located. If all other authors were from one hospital, and the first author exclusively was not, then the hospital of the last author defined the country. If an article addressed several surgical approaches, the data of each treatment strategy were managed separately^10^. Because the statistical measures were not standardized, mean and median reports were treated equally to take into account the cluster of affected patients who were young adults^10^. Data presented as range of follow-up times was managed by employing the center of the given time^10^.

Recurrence rates in each study were then associated with the reported follow-up time. Individual patients were statistically simulated to enable an analogy across all data^10^. Cochrane analysis and I2 calculation with Chi^2^ tests were employed to examine heterogeneity of the included data^10^.

Statistical analysis and figure generation were completed with the software “R” (version 3.1.0, R-studio framework version 0.98.982). Two-tailed statistical tests were performed^10^. Kaplan-Meier curves of recurrence-free outcome, including pointwise 95% confidence intervals (CI), were generated with ‘survival’ in “R” (version 2.40–1) and implemented in the R package for each therapeutic group in each geographic region.

Data with unknown geographical origin were excluded. The United States, Turkey, Italy, Germany and Greece were defined as single countries; other countries were grouped into regions (Northern Europe, the Mediterranean, North America, i.e. USA and Canada, Australia/New Zealand, Indo-Arabia, Asia and South America) to obtain sufficient sample sizes.

### Category

Post hoc analyses of data for a systematic review and meta-analysis, no publication before. No submission in parallel. No full or partial presentation at a meeting or podium or conference.

### Ethics

This article does not contain any studies with human participants. Therefore, no informed consent had to be obtained prior to preparation of the current manuscript.

### Presentation

The manuscript has not been submitted elsewhere in parallel and has not been published previously. Some of the data were presented at the International Pilonidal Sinus Disease Conference in Berlin on Sept 23, 2017 and at the 2nd International Pilonidal Sinus Conference in Vienna on Sept 28th 2019.

## Supplementary information

Supplementary Information

## Data Availability

All data and calculations are available to readers upon request to the corresponding author.
