# Supplementary material for: Impact of geography and surgical approach on recurrence in global pilonidal sinus disease
Source: Sci Rep. 2019 Oct 22;9:15111. doi: 10.1038/s41598-019-51159-z (PMC6805955; doi:10.1038/s41598-019-51159-z)
Supplement: Supplementary file 1 — Supplementary Information [file 41598_2019_51159_MOESM1_ESM.pdf]

Doll D, Orlik A, Maier K, Kauf P, Schmid M, Diekmann M, Vogt AP, Stauffer VK, Luedi MM:

Impact of geography and surgical approach on recurrence in global pilonidal sinus disease

Supplemental Figures and Tables

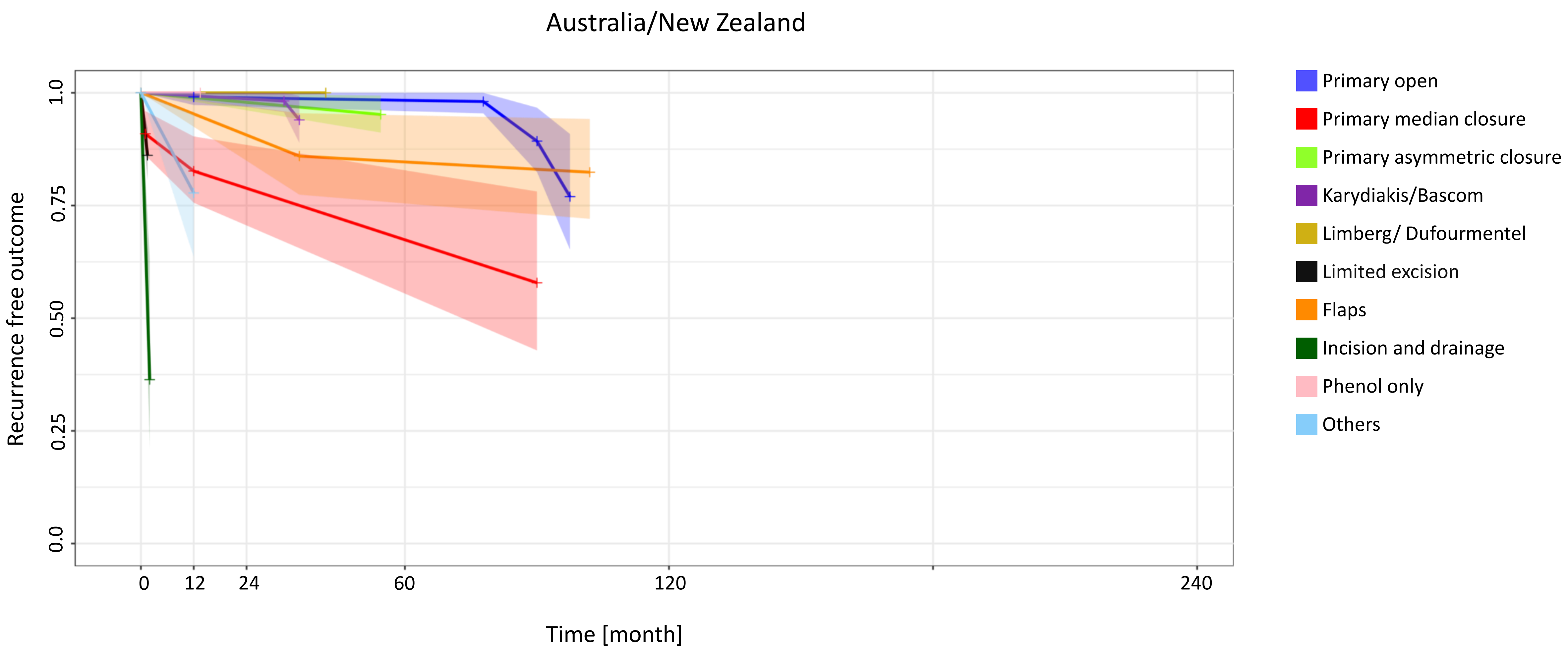

Supplemental Figure 1. Australia / New Zealand: Kaplan-Meier estimator depicting recurrence-free outcome of the study population as a function of follow-up time. The data used include all available studies from Italy. 95% confidence intervals are shown by shaded lines. Number of patients and CI 95% data included in the analysis are shown in Supplemental Table 1. Shaded lines indicate 95% confidence intervals

Supplemental Table 1: Recurrence rates in percent as a function of follow-up time of patients and all available surgical approaches applied in Australia and New Zealand. Number of patients included in the analysis and the according 95% CI are indicated at 0, 12, 24, 60, and 120 months. All used citations for the analysis are shown in the right column.

**Australia + New Zealand**	**12 months**		**24 months**		**60 months**		**120 months**		**Citations**
**Surgical method (total patients included)**	**RR in % (95% CI)**	**Nr. at risk**	**RR in % (95% CI)**	**Nr. at risk**	**RR in % (95% CI)**	**Nr. at risk**	**RR in % (95% CI)**	**Nr. at risk**	
Primary open (108)	0.9 (0.0-2.8)	108	1.1* (0.0-3.1)	106*	1.7* (0.0-4.2)	101*	NA	NA	114,624,625
Primary median closure (132)	17.4 (8.3-26.4)	77	21.2* (8.8-33.6)	68*	32.6* (10.3-55.0)	42*	NA	NA	205,624,625
Primary asymmetric closure (104)	1.1* (0.1-2.0)	104*	2.1* (0.2-4.1)	104*	NA	NA	NA	NA	242
Karydiakis/Bascom (110)	0.7* (0.0-1.6)	110*	1.3* (0.0-3.3)	110*	NA	NA	NA	NA	270,626
Limberg/Dufourmentel (29)	0.0* (0.0-0.0)	29*	0.0* (0.0-0.0)	29*	NA	NA	NA	NA	627
Limited excision (137)	NA	NA	NA	NA	NA	NA	NA	NA	205
Flaps (57)	4.7* (1.1-8.2)	57*	9.4* (2.2-16.5)	57*	15.3* (3.6-27.1)	45*	NA	NA	460,628
Incision and drainage (22)	NA	NA	NA	NA	NA	NA	NA	NA	205
Phenol only (20)	0.0* (0.0-0.0)	20*	NA	NA	NA	NA	NA	NA	629
Others (27)	22.2 (1.6-42.8)	27	NA	NA	NA	NA	NA	NA	625

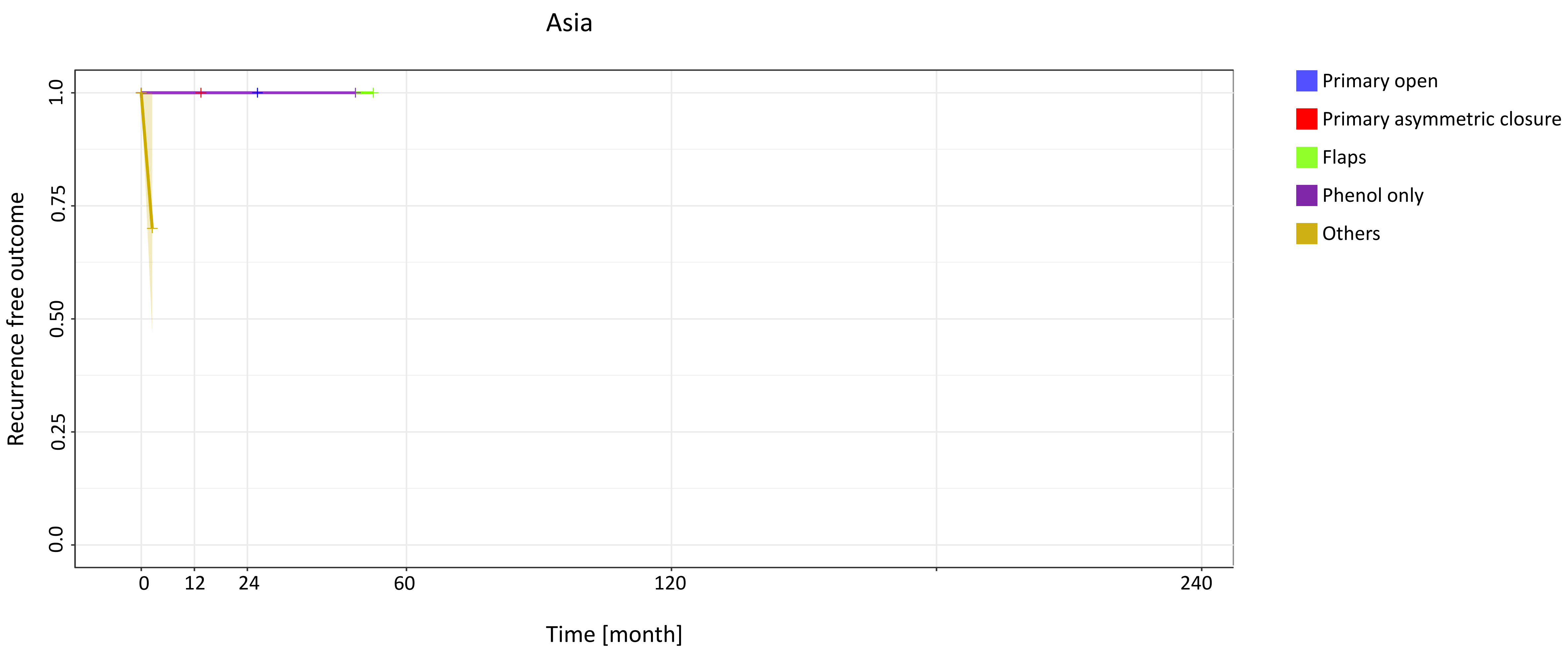

Supplemental Figure 2. Asia: Kaplan-Meier estimator depicting recurrence-free outcome of the study population as a function of follow-up time. The data used include all available studies from Italy. 95% confidence intervals are shown by shaded lines. Number of patients and CI 95% data included in the analysis are shown in Supplemental Table 2. Shaded lines indicate 95% confidence intervals

Supplemental Table 2: Recurrence rates in percent as a function of follow-up time of patients and all available surgical approaches applied in Asia. Number of patients included in the analysis and the according 95% CI are indicated at 0, 12, 24, 60, and 120 months. All used citations for the analysis are shown in the right column.

**Asia**	**12 months**		**24 months**		**60 months**		**120 months**		**Citations**
**Surgical method (total patients included)**	**RR in % (95% CI)**	**Nr. at risk**	**RR in % (95% CI)**	**Nr. at risk**	**RR in % (95% CI)**	**Nr. at risk**	**RR in % (95% CI)**	**Nr. at risk**	
Primary open (9)	0.0* (0.0-0.0)	9*	0.0* (0.0-0.0)	9*	NA	NA	NA	NA	106
Primary asymmetric closure (17)	0.0* (0.0-0.0)	17*	NA	NA	NA	NA	NA	NA	248
Flaps (7)	0.0* (0.0-0.0)	7*	0.0* (0.0-0.0)	7*	NA	NA	NA	NA	472
Phenol only (32)	0.0* (0.0-0.0)	32*	0.0* (0.0-0.0)	32*	NA	NA	NA	NA	630
Others (10)	NA	NA	NA	NA	NA	NA	NA	NA	512

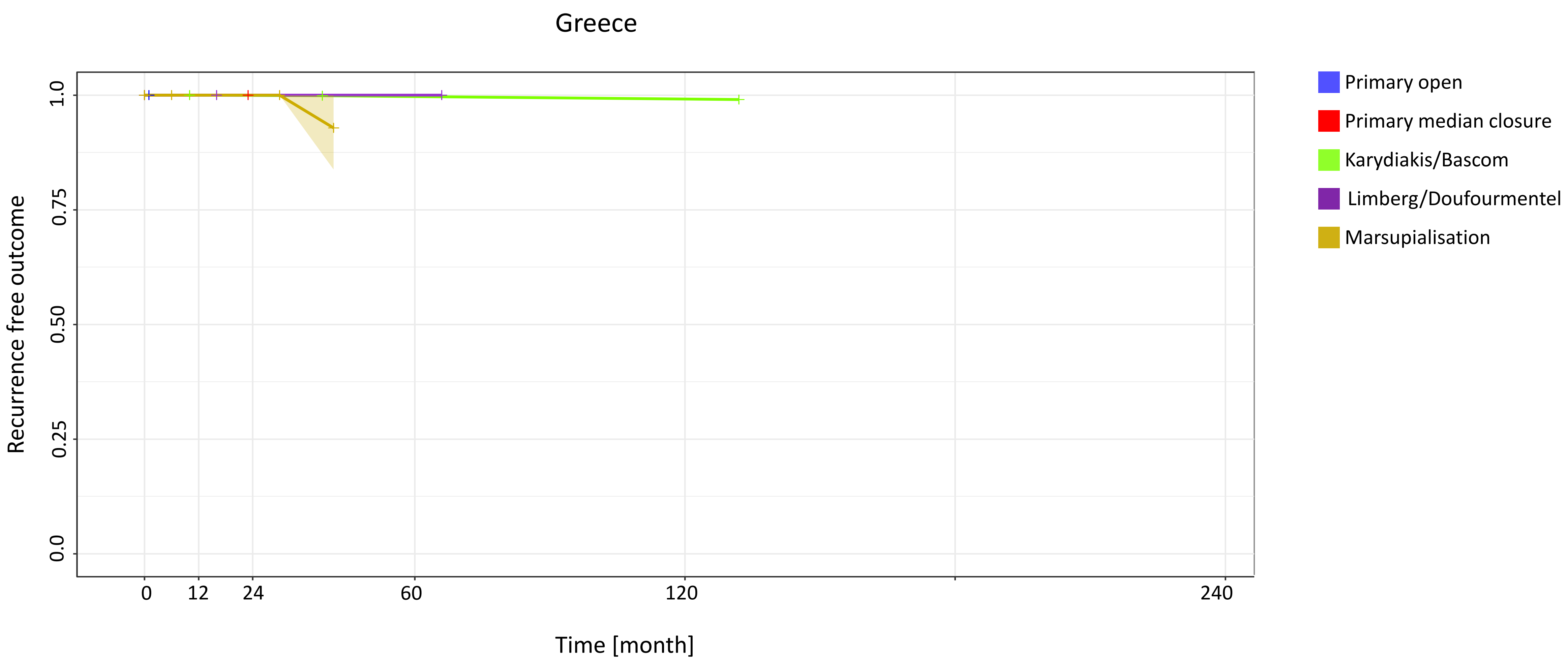

Supplemental Figure 3. Greece: Kaplan-Meier estimator depicting recurrence-free outcome of the study population as a function of follow-up time. The data used include all available studies from Italy. 95% confidence intervals are shown by shaded lines. Number of patients and CI 95% data included in the analysis are shown in Supplemental Table 3.

Supplemental Table 3: Recurrence rates in percent as a function of follow-up time of patients and all available surgical approaches applied in Greece. Number of patients included in the analysis and the according 95% CI are indicated at 0, 12, 24, 60, and 120 months. All used citations for the analysis are shown in the right column

**Greece**	**12 months**		**24 months**		**60 months**		**120 months**		**Citations**
**Surgical method (total patients included)**	**RR in % (95% CI)**	**Nr. at risk**	**RR in % (95% CI)**	**Nr. at risk**	**RR in % (95% CI)**	**Nr. at risk**	**RR in % (95% CI)**	**Nr. at risk**	
Primary open (52)	NA	NA	NA	NA	NA	NA	NA	NA	40
Primary median closure (43)	0.0* (0.0-0.0)	43*	NA	NA	NA	NA	NA	NA	631
Karydiakis/Bascom (7240)	0.0* (0.0-0.0)	7238*	0.1* (0.0-0.1)	7229*	0.3* (0.2-0.4)	7067*	0.9* (0.6-1.1)	NA	278,632,633
Limberg/Dufourmentel (142)	0.0* (0.0-0.0)	142*	0.0* (0.0-0.0)	137*	0.0* (0.0-0.0)	114*	NA	NA	364,634
Marsupialisation (77)	0.0* (0.0-0.0)	77	0.0* (0.0-0.0)	55*	NA	NA	NA	NA	408,635
